# Establishment and Validation of a Method for the Identification of Recessive Mastitis Resistance Genes in Dairy Cows

**DOI:** 10.3390/genes16050485

**Published:** 2025-04-25

**Authors:** Wei Zheng, Pei Wu, Mengting Zhu, Yaseen Ullah, Zongsheng Zhao, Shaoqi Cao, Guang Li, Sihai Ou, Kaibing He, Ye Xu

**Affiliations:** 1 College of Animal Science and Technology, Shihezi University, Shihezi 832003, China; zhengwei890801@shzu.edu.cn (W.Z.);; 2 College of Animal Science, Xinjiang Agricultural University, Urumqi 830052, China; 3 Xinjiang Uygur Autonomous Region Animal Husbandry General Station, Urumqi 830052, China; 4 Animal Husbandry and Fishery Development Service Center of the 8th Division, Shihezi, Xinjiang Production and Construction Corps, Shihezi 832003, China

**Keywords:** occult mastitis, Kompetitive allele-specific PCR, MPVA, SNP, Holstein dairy cows

## Abstract

Background/Objectives: The resistance to occult mastitis in dairy cows is a multifaceted trait influenced by a variety of genetic and environmental factors, posing significant challenges to its prevention and treatment. Methods: In this study, a cohort of 389 Holstein dairy cows was selected for investigation. The genes NOD2, CXCR1, SPP1 and LF, which are implicated in resistance to occult mastitis, were genotyped utilizing the efficient and cost-effective Kompetitive Allele-Specific PCR (KASP) technology. Additionally, the study analyzed the association between various single nucleotide polymorphisms (SNPs) and the somatic cell score in Holstein dairy cows. Multi-locus penetrance variance analysis (MPVA) analysis was also conducted to assess the resistance of different genotypic combinations to recessive mastitis in dairy cows. A genotyping kit for occult mastitis resistance was developed. Subsequently, 300 Holstein cows were randomly selected to evaluate the accuracy of the kit’s classification and resistance detection. Results: The findings revealed that the most effective genotype combination was SPP1(AA)-CXCR1(CC)-NOD2(CA)-LF(GA). Upon verification, the genotyping kit for recessive mastitis resistance in dairy cows exhibited an accuracy rate of 100% for individual genotyping and 95.90% for resistance detection. Conclusions: From the perspective of disease resistance genetics, this study lays a foundation for the precise management of dairy cow herds. It enables the early identification and removal of individuals susceptible to subclinical mastitis, thereby improving the overall quality of the cattle population.

## 1. Introduction

Mastitis is a prevalent global disease that poses significant risks to the health and welfare of Holstein dairy cows [1,2,3] and represents the most costly disease within the dairy industry [4,5,6,7,8]. Both clinical mastitis (CM) and occult mastitis [9,10,11,12] contribute to decreased milk production. The elevation in somatic cell count and alterations in milk composition [13] severely impact the lactational performance of dairy cows. Particularly in the case of occult Mastitis, which lacks overt clinical symptoms, an increased somatic cell count can be detected, and the condition is susceptible to recurrent infections, resulting in substantial economic losses for the dairy cattle breeding industry [14,15]. The etiology of mastitis is multifactorial, predominantly influenced by environmental conditions, feeding practices, management strategies, and individual genetic factors [16]. Currently, research on mastitis in dairy cows predominantly emphasizes the use of biological agents and herbal medicines as alternatives to antibiotics [17]. Studies have explored probiotic interventions and the regulation of the host’s intestinal microbiota for the prevention and treatment of mastitis [18]. Additionally, the pathogenic mechanisms of mastitis have been investigated using pathogenic microorganisms and omics techniques [19,20,21]. However, the overall effectiveness of these approaches has been relatively limited. Consequently, to more effectively mitigate the adverse effects of mastitis on the dairy industry, a promising new avenue of research involves the identification of genetic resistance markers for mastitis in dairy cows [22,23].

Previous research has demonstrated that the susceptibility or resistance of individual dairy cows to mastitis is predominantly influenced by genetic factors [24,25,26]. Key indicators of this susceptibility and resistance include somatic cell count (SCC) and somatic cell score (SCS) [27,28,29,30,31], with the latter showing a strong correlation with single nucleotide polymorphisms (SNPs) at various gene loci in dairy cows [32,33]. By examining the SNPs of candidate genes and the genetic markers associated with mastitis resistance or susceptibility [34,35,36], it is possible to achieve the objective of mastitis prevention through the lens of disease-resistant genetics [37].

Various methods exist for genotyping based on single nucleotide polymorphisms (SNPs), with single-strand conformation polymorphism analysis (PCR-SSCP) [38] and polymerase chain reaction-restriction fragment length polymorphism (PCR-RFLP) [39] being among the most commonly employed. Nevertheless, these techniques are often time-intensive and slow, making them more suitable for fundamental research with relatively small sample sizes. In the post-genomic era, the third-generation molecular marker SNP emerged, leading to the development of Kompetitive Allele-Specific PCR (KASP) genotyping technology by LGC Company. This technology allows for precise biallelic determination of SNPs and insertions/deletions (INDELs) at specific loci. Currently, KASP is extensively utilized in genetic improvement research in both animals and crops. In the domains of animal science, botany, and medicine, KASP technology enables rapid detection of SNPs linked to target genes and precise identification of gene mutations caused by individual base changes [40,41].

Mastitis is a complex trait influenced by multiple genes [1,42,43]. Current research predominantly focuses on the association between individual genes or single nucleotide polymorphisms (SNPs) and mastitis, with relatively fewer studies investigating the aggregation of multiple genes. The existing literature suggests that breeding strategies based on the aggregation of multiple genes are both theoretically and practically feasible. Polygene aggregation analysis enables the integration of multiple advantageous genes. In this context, the present study aims to identify the optimal genotype combinations for resistance to recessive mastitis in dairy cows through multi-locus penetrance variance analysis (MPVA) [44]. This approach seeks to identify individuals with enhanced resistance to recessive mastitis, thereby laying the groundwork for early disease-resistant breeding in dairy cows and improving overall herd quality.

The research group initially conducted experimental studies on nine loci associated with mastitis. Ultimately, four locis-NOD2c.4500, CXCR1+777, SPP1-1301 and LF-190 exhibiting significant differences were selected for further experimentation.

Although Secreted Phosphoprotein 1 (SPP1) is expressed in various organs, its highest expression is observed in the mammary gland [45]. The estimated concentration of SPP1 secreted in milk is 18 mg per liter [46], while its concentration in colostrum is ten times higher. Some studies suggest that the high abundance of SPP1 in milk may be linked to its role in neonatal immunity [47]. Genetic variation in bovine SPP1 influences the secretion of osteopontin in milk throughout lactation [48]. Additionally, its expression increases during mammary gland infections, enhancing cell-mediated immunity.

CXC-chemokine receptor 1 (CXCR1) primarily encodes pro-inflammatory cytokines and has been shown to play a crucial role in bacterial detection and elimination. It is expressed on the surface of neutrophils, which are essential for their migration into the mammary gland and the resolution of bacterial infections [49]. The CXCR1 gene exhibits high polymorphism, with up to 36 single nucleotide polymorphisms (SNPs) identified within the coding region and adjacent sequences of Holstein cows [50]. Several of these SNPs have been associated with mastitis resistance in various sample groups of Holstein cows [50,51].

Nucleotide-binding oligomerization domain protein 2 (NOD2), a member of the N-terminal caspase recruitment domain intracellular protein family has been extensively studied in the context of the human immune system [52]. The primary functions of the NOD2 gene/protein include defending against microbial infections, regulating inflammatory processes, and induction of cell apoptosis. It enhances autophagy, processes damaged organelles and provides cellular protection. NOD2 is also implicated in the immune response to various bacteria, including *Staphylococcus aureus*, *Escherichia coli*, and *Streptococcus pneumoniae*. Recently, the NOD2 gene was identified in bovine mammary glands following intramammary infection with *Staphylococcus aureus* [53]. Research conducted by Wang et al. demonstrates that both *Staphylococcus aureus* and *Escherichia coli* can stimulate the expression of the NOD2 gene in mammary epithelial cells [54].

Lactoferrin (Lf) is a non-heme iron-binding protein that belongs to the transferrin family. The bovine lactoferrin gene is located on chromosome 22 and comprises 17 exons, spanning approximately 34.5 kilobase pairs (KBP) of genomic DNA [55]. Molenaar et al. [56] confirmed that the LF gene is highly expressed in breast tissue affected by mastitis and that the lactoferrin gene may serve as a resistance gene against mastitis in dairy cows. Lactoferrin plays a crucial role in combating breast infections, including its involvement in the complement system within infected breast tissue, the mechanisms underlying inflammatory responses, and the behavior of immune cells. Ohashi et al. [57] demonstrated that the lactoferrin gene can confer resistance to mastitis infections in dairy cows by inhibiting the protease activity of viruses and bacteria. Furthermore, McDermott and Suzuki [58,59] demonstrated lactoferrin’s involvement in and its ability to enhance immune regulation within the body. Ryman et al. [60] showed that lactoferrin binds to ferric iron ions, thereby inhibiting bacterial proliferation through its antibacterial effects. In addition to these properties, lactoferrin exhibits direct bactericidal effects against specific mastitis-causing pathogens and is recognized for its role in maintaining normal lymphocyte and macrophage function. Building upon the previous research conducted by our group, this study identified four gene loci significantly associated with resistance to recessive mastitis. We utilized Kompetitive Allele-Specific PCR (KASP) technology for genotyping, examining the genetic characteristics in relation to somatic cell scores. Additionally, a multi-locus penetrance variance analysis was conducted on the significantly associated SNP loci, culminating in the development of a rapid genomic detection kit for recessive mastitis resistance in dairy cows using KASP technology. This research aims to provide a theoretical foundation for molecular breeding strategies designed to enhance resistance to recessive mastitis through multi-gene aggregation. Furthermore, it offers management recommendations for the precise feeding of cattle herds affected by recessive mastitis and holds substantial practical significance for the early selection and breeding of individuals exhibiting resistance to recessive mastitis in dairy cows.

## 2. Materials and Methods

### 2.1. Ethics Approval

The experimental procedures and study design involving animals in the present research were sanctioned by the Biology Ethics Committee of Shihezi University (Shihezi, China) under approval number A2024-442. Blood samples were obtained from the animals by qualified veterinarians. All research activities conducted in this study adhered to Directive 2010/63/EU, which pertains to the protection of animals utilized for scientific purposes.

### 2.2. Animals and Samples Collection

In this study, a cohort of randomly selected Chinese Holstein dairy cows was maintained at Shihezi Farm in Xinjiang, China (coordinates: 116°23′28.841″ E, 39°54′19.417″ N). The sample comprised 689 healthy dairy cows in their first, second, or third lactation, with an average body weight of 480 ± 50 kg. Of these, 389 cows were utilized for an association analysis involving Kompetitive Allele-Specific PCR (KASP) genotyping and somatic cell typing to assess resistance to occult mastitis. An additional 300 cows were employed in the development and evaluation of a KASP genotyping kit for occult mastitis. The entire herd participated in the Dairy Herd Improvement (DHI) program, with data recorded on a monthly basis.

Milking was conducted thrice daily in a “herringbone” milking parlor (2 sides × 14 stalls) using the Yimu Cloud Digital Dairy Mobile Platform^®^ for farm management. Throughout the research period, the cows were housed on straw bedding, with each animal allocated 10 m^2^ in the resting area and unrestricted access to an outdoor enclosure, ensuring a dry and clean environment. The cows were grouped in clusters of 40 to 50 based on their lactation stage and milk yield. Furthermore, all dairy cows are fed twice daily with a diet that includes dry roughage, such as wheat straw and green feed, contingent upon seasonal availability while maintaining unrestricted access to water. The feeding regimen for dairy cows is adjusted according to seasonal variations. Blood samples were collected by trained veterinarians, with 5 mL of blood drawn from the jugular vein of each cow and transferred into vacuum collection tubes containing the anticoagulant EDTA-K_2_. These samples are subsequently stored at −20 °C for further analysis.

### 2.3. Blood DNA Extraction and Detection

Genomic DNA was extracted following the instructions provided by the blood genomic DNA extraction kit (TIANGEN, Beijing, China) from the total blood of experimental cows. The concentration and quality of the extracted DNA were assessed using an ultramicrospectrophotometer (Thermo Fisher Scientific, Waltham, MA, USA) and 2% agarose gel electrophoresis (Bio-Rad, Hercules, CA, USA). All DNA samples were diluted to a uniform concentration, with an equivalent of 50 ng of DNA per sample utilized for subsequent experimental studies.

### 2.4. KASP Genotyping Design

In previous laboratory studies, four genes (NOD, CXCR1, SPP1, and LF) and their SNP loci were found to be significantly associated with occult mastitis resistance in dairy cows. The NOD2 C.4500 allele contains a C/A mutation at position 19119273 bp (NC_037329.1) on bovine chromosome 18. The CXCR1+777 allele exhibits a G/C mutation at position 1062016092 bp (NC_037345.1) on bovine chromosome 2. The SPP1-1301 allele features a G/A mutation at position 36700265 bp (NC_037333.1) on bovine chromosome 6. Lastly, the LF-190 allele presents a G/A mutation at position 75284221 bp (NC_037332.1) on bovine chromosome 5.

In this study, the sequence characteristics of the alleles NOD2c.4500, CXCR1+777, SPP1-1303 and LF-190 were utilized to design KASP-specific primers (see Table 1). The KASP primers for each SNP, which consist of two allele-specific forward primers and a common reverse primer, were designed and genotyped by LGC Genomics (LGC, Hoddesdon, Herts, UK). Mixed test preparation and PCR amplification were conducted following user guidelines and manuals provided by LGC Genomics (LGC, Hoddesdon, Herts, UK). The concentration of DNA samples was measured using an ultramicrospectrophotometer (Thermo Scientific, Waltham, MA, USA) and diluted as necessary. Genotyping was carried out on a 96-well plate with a reaction volume of 10 µL, comprising 4 µL of 2× KASP Master Mix (LGC, UK), 0.15 µL of each of the two forward primers, 0.4 µL of the reverse primer, and 1 µL of genomic DNA (at a concentration of 5–50 ng/µL), supplemented with ddH_2_O to achieve a final volume of 10 µL. In the negative control reaction, DNA samples were replaced with an equivalent volume of ddH_2_O.

The sealing plate was then covered with Micro-Amp Optical adhesive film (Thermo Fisher Scientific, Waltham, MA, USA) and centrifuged (Thermo Fisher Scientific, Waltham, MA, USA) at 5000× *g* for 30 s. KASP genotyping was performed using the Step One Plus^TM^ real-time PCR system (Thermo Fisher Scientific, Waltham, MA, USA) under the following cycling conditions: a pre-read phase of 60 s at 30 °C, followed by 10 min at 95 °C, then 10 cycles of 95 °C for 20 s and 61 °C (with a 0.6 °C reduction per cycle) for 45 s, followed by 38 cycles of 95 °C for 20 s and 55 °C for 45 s, concluding with a post-read phase of 60 s at 25 °C.

### 2.5. Design of Multi-Locus Penetrance Variance Analysis (MPVA)

MPVA is a method for analyzing genes and gene interactions, proposed by Sun et al. [61], primarily utilized for investigating disease susceptibility interactions. The fundamental formula of the MPVA model is as follows:

(1) $$P\left(D/G_{i}\right)=\frac{P\left(G_{i}/D\right)P\left(D\right)}{P\left(G_{i}\right)}\;\;\;\;\;\;\;\;\;\;\;\;i\;\epsilon \left\{1,\;2,\;\ldots m\right\}\;$$

In this formula, *D* and *G_i_* denote mastitis and the corresponding genotypes, respectively. *P*(*D*/*G_i_*) represents the probability of mastitis occurring in cows with the *G_i_* genotype, while *P*(*G_i_*/*D*) indicates the probability of the *G_i_* genotype in cows diagnosed with mastitis. Additionally, *P*(*D*) signifies the incidence of mastitis within dairy herds, and *P*(*G_i_*) refers to the probability of the *G_i_* genotype among dairy herds.

If a genetic correlation exists between the multi-locus genotype *G_i_* and mastitis, then *P*(*D*/*G_i_*) is not equal to *P*(*D*). Furthermore, the mean squared error (MSE) of *P*(*D*/*G_i_*) can be expressed as follows:

(2) $$MSE=\sum_{i=1}^{m}P(G_{i}){\left[P\left(D/G_{i}\right)-P\left(D\right)\right]}^{2}\;$$

MSE serves as an indicator of the correlation between multi-locus genotypes and mastitis, with higher values indicating a stronger correlation. Zhang Yaqin [44] demonstrated that the susceptibility of individual dairy cows to recessive mastitis decreased as the MSE value of the genotype combination decreased, thereby enhancing disease resistance. Consequently, the optimal genotype combination for combating recessive mastitis in dairy cows can be identified by selecting the genotype combination with the lowest MSE value.

### 2.6. Statistical Analysis

The genotype frequency, allele frequency, polymorphism information content (*PIC*), homozygosity (*Ho*), heterozygosity (*He*), and effective allele number (*Ne*) for the following genes were calculated using SPSS (Version 29.0; IBM, Armonk, NY, USA): NOD2 c.4500, CXCR1+777, SPP1-1303 and LF-190. The least squares analysis method in SAS (version 9.4; SAS Institute, Cary, NC, USA) was employed to investigate the association between SNP sites and somatic cell score. The model is defined as follows:

(3) $$Y_{jklmn}=\mu +L_{j}+S_{k}+N_{l}+G_{m}+e_{jklmn}\;$$

Among the variables, *Y_jklmn_* represents the observed value of individual traits; *μ* denotes the population mean; *L*_j_ indicates the fixed effect of the *j* parity; S_k_ reflects the age effect; *N_l_* accounts for the seasonal effect; *G_m_* represents the fixed effect of the site; and *e_jklmn_* is the standard error.

### 2.7. Construction and Evaluation of KASP Genotyping Kit for Occult Mastitis in Dairy Cows

#### 2.7.1. Test Animals

Three hundred cows from another experimental group with similar body weights and good physical conditions in a large dairy farm in the Shihezi area were randomly selected as the experimental subjects. Complete DHI data for nearly 3 years during lactation, as well as monthly collection of tail vein blood, heparin sodium anticoagulation and DHI data.

#### 2.7.2. The Determination of Resistance to Occult Mastitis in Dairy Cows

The somatic cell count (SCC) in Dairy Herd Improvement (DHI) records was utilized to determine cow resistance indirectly. The specific criteria were as follows: if the average SCC over the entire lactation period was less than 200,000 cells/mL, the cow was classified as highly resistant. Conversely, if the average SCC exceeded 500,000 cells/mL, the cow was deemed susceptible. Cows with an average SCC between 200,000 and 500,000 cells/mL were categorized as moderately resistant. The criteria for analyzing DHI data included a lactation period ranging from 7 to 360 days postpartum, a daily milk yield of 2 to 60 kg, a daily milk fat percentage of 2% to 7%, a daily milk protein percentage of 2% to 6%, and a total milk yield of 1000 to 18,000 kg over 305 days. Additionally, the 305-day milk fat yield ranged from 34 to 555 kg, while the 305-day milk protein yield varied from 29 to 700 kg.

#### 2.7.3. Construction of KASP Genotyping Kit and Evaluation of Detection Effects

(1) Composition and storage condition of the KASP genotyping kit

The composition of the KASP genotyping kit for occult mastitis in dairy cows is shown in Table 2. The kit should be stored at −20 °C, protected from light, and should not be stored long-term at 4 °C or room temperature.

(2) Operation procedure

DNA extraction from the sample to be tested is performed using the blood genome extraction kit (Tiangen, Beijing, China).

Thaw and gently mix upstream primer F1, upstream primer F2, universal downstream primer, and the 2× KASP Master mix included in the kit. Ensure that the reagents in the tube are concentrated at the bottom, and keep the mixture on ice.

Prepare the PCR reaction mixture on ice according to Table 3. The entire reaction process should be conducted away from light. After adding the reaction mixture, seal the container immediately with an optically opaque cover to prevent evaporation.

(3) The PCR reaction system is presented in Table 4. After the reaction is completed, the PCR plate can be stored at 4 °C for up to one week, during which the fluorescence signal remains relatively stable. Additional cycles or data re-reads can be performed within this one-week period.

## 3. Results

### 3.1. KASP Genotyping

**Figure 1:**
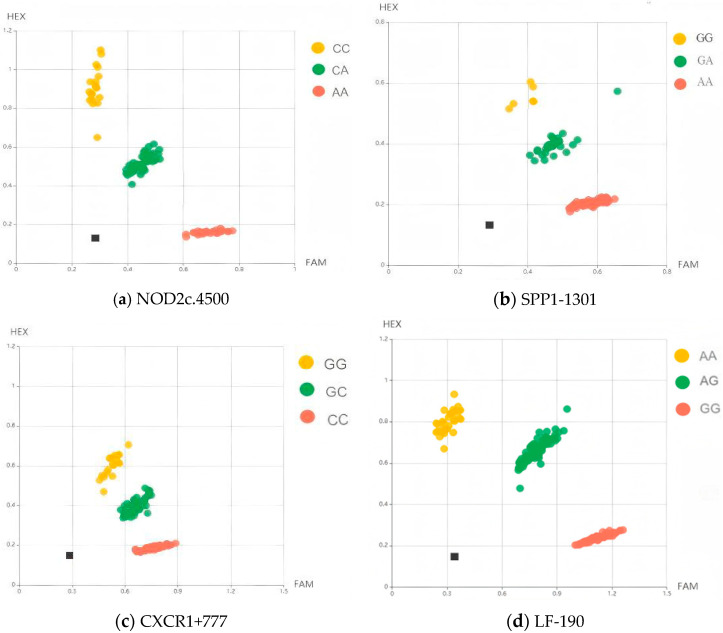
Graphics of KASP test results show the genotypes of four genes: NOD2c.4500 (**a**), SPP1-1301 (**b**), CXCR1+777 (**c**) and LF-190 (**d**). Red and yellow dots correspond to homozygous genotypes, green dots correspond to heterozygous genotypes, and black dots correspond to non-template control samples.

The KASP genotyping technique was employed to detect the recessive mastitis resistance genome in 389 dairy cows. The results demonstrated that the previously developed KASP-specific primers and detection methods were effective in distinguishing the various genotypes of NOD2, SPP1, CXCR1 and LF (Figure 1). At the conclusion of the PCR amplification cycle, fluorescence values were recorded by an enzyme-labeled instrument at room temperature. The LC480 Software v1.5.1 was utilized for analyzing the typing results. Genotyping data were visualized as a cluster plot using the SNP viewer software (version 1.99, Hoddesdon, UK). Homozygous individuals were identified through a 5′-terminally linked FAM fluorescent label sequence, which generated red aggregation of fluorescence signals along the X-axis. Genotype samples exhibiting red fluorescence were homozygous for the FAM-labeled alleles, whereas homozygous individuals identified by a 5′-terminally linked HEX fluorescent label sequence produced yellow fluorescence signal aggregation along the Y-axis. The yellow genotype samples represented homozygous mutants for the HEX-labeled alleles, while samples marked in green indicated hybrids. The genotype data of each experimental animal were derived for subsequent statistical analysis.

### 3.2. Genetic Diversity Analysis of NOD2c.4500, CXCR1+777, SPP1-1303, and LF-190 Genes

#### 3.2.1. Analysis of Genetic Characteristics of Different SNPs in Holstein Dairy Cows

**Figure 2:**
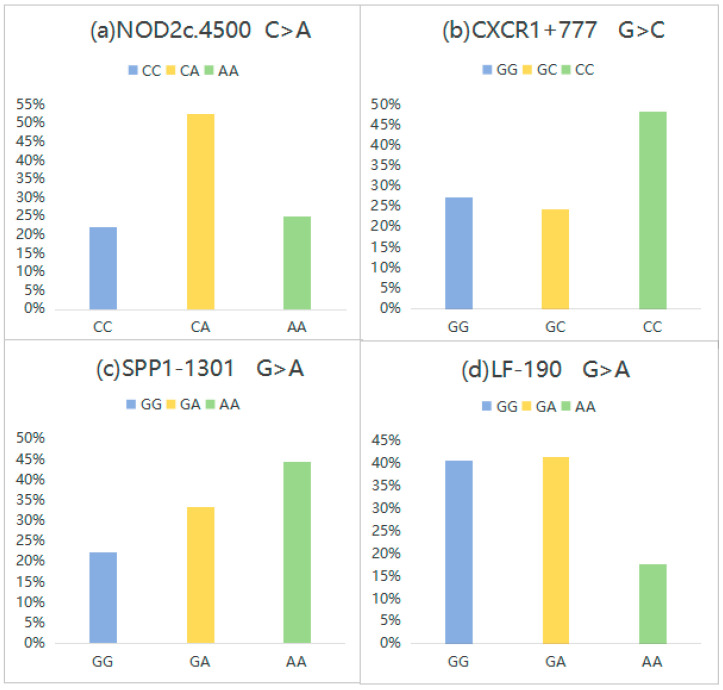
(**a**) Genotyping results of NOD2c4500 C>A, (**b**) Genotyping results of CXCR1+777 G>C, (**c**) Genotyping results of SPP1-1301 G>A, (**d**) Genotyping results of LF-190 G>A.

In this part, the genetic characteristics of SNP loci across various genes in Holstein dairy cows were analyzed with the result of the distribution of genotyping by gene loci NOD2c4500, CXCR1+777, SPP1-1301 and LF-190 shown in Figure 2, and the results of genotype frequencies, allele frequencies and main diversity indices for the polymorphic SNPs saw in Table A1. The medium dominant alleles for NOD2c.4500 C>A, CXCR1+777 G>C, SPP1-1301 G>A, and LF-190G>A were CA, GG, AA, and GA, respectively, with the corresponding dominant genes being A, G, A, and G. All four loci were found to be in a Hardy–Weinberg equilibrium state (*p* > 0.05). Furthermore, SPP1-1301 G>A exhibited low polymorphism (*PIC* < 0.25), NOD2c.4500 C>A, CXCR1+777 G>C, and LF-190 G>A displayed moderate polymorphism (0.25 < *PIC* < 0.5). 

#### 3.2.2. Association Analysis of Different Genes and SNPs on Somatic Cell Score of Holstein Dairy Cows

As shown in Table A2, the individual cell scores for the NOD2c.4500 C>A loci with the CA genotype were significantly lower than those of the CC and AA genotypes (*p* < 0.05). Similarly, the individual cell scores for the CXCR1+777 G>C loci were significantly lower than those of the GG and CG genotypes (*p* < 0.05). Furthermore, the individual cell score for the CC genotype at the G>A locus in SPP1-1301 was significantly lower than that of the GG and GA genotypes (*p* < 0.05). Additionally, the individual cell score for the GA genotype at the G>A locus in LF-190 was significantly lower than that of the GG and AA genotypes (*p* < 0.05).

#### 3.2.3. Mufti-Locus Penetrance Variance Analysis

In the multi-seat explicit variance analysis of the NOD2, CXCR1, SPP1, and LF genes and their loci, a substitution test was performed. Multi-seat combinations were selected based on a *p* > 0.5, identifying SPP1-1301-CXCR1+777-NOD2 c.4500-LF-190 as the optimal multi-seat combination. The mean squared error (MSE) value for the genotype combination SPP1(AA)-CXCR1(CC)-NOD2(CA)-LF(GA) was found to be 1.01 × 10^−4^, while the mean ± standard deviation of the somatic cell count (SCC) was 17.45 ± 50.2 Thousand per milliliter (Table A3). These results suggest that the genotype combination SPP1(AA)-CXCR1(CC)-NOD2(CA)-LF(GA) may serve as the most effective genotype combination.

### 3.3. Validation of KASP Genotyping Kit for Occult Mastitis in Dairy Cows

#### 3.3.1. KASP Genotypes and Classification of Resistance to Occult Mastitis for 300 Dairy Cows

**Figure 3:**
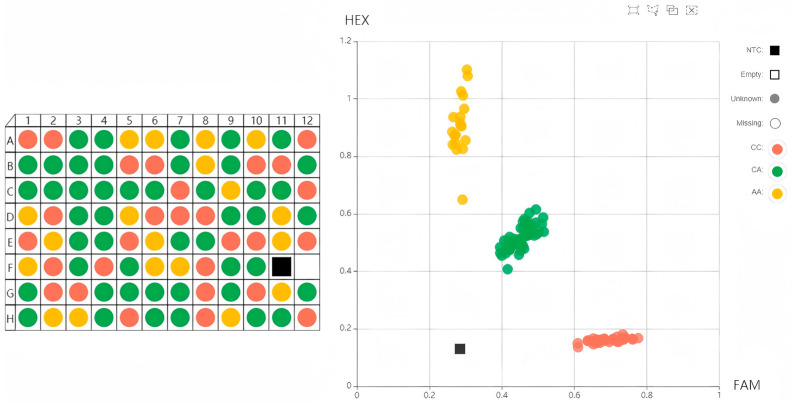
The genotyping results for NOD2c.4500, CXCR1+777, SPP1-1301, and LF-190 were obtained using KASP in a sample of 300 Holstein dairy cattle.

At the end of the PCR amplification cycle, fluorescence values were recorded by an enzyme-labeled instrument at room temperature. Genotyping data were visualized as a cluster plot using the SNP viewer software (version 1.99, Hoddesdon, UK) (Figure 3). Genotypes marked in yellow indicate homozygosity for HEX-labeled alleles, genotypes marked in red indicate homozygosity for FAM-labeled alleles, samples marked in green represent heterozygosity, and black dots signify blank controls.

Based on the correlation between genotypes and somatic cell score discussed in Section 3.2.2, as well as the size of the mean square error (MSE) outlined in Section 3.2.3, different genotypes can be categorized into susceptible individuals. Dairy cows with MSE values exceeding 1 × 10^−1^ or possessing only one or no resistance genotypes are classified as susceptible. Cows with MSE values ranging from 1 × 10^−2^ to 1 × 10^−1^, or those with only two resistance genotypes, are classified as moderately resistant. Conversely, cows with MSE values below 1 × 10^−2^ or containing three or more resistance genotypes are categorized as highly resistant.

Based on the average somatic cell count (SCC) results over the entire lactation period from the Dairy Herd Improvement (DHI) data, a correlation analysis was conducted between genotype and somatic cell score (as detailed in Table A2). This analysis, alongside the mean squared error (MSE) values reported in Table 5, facilitated the classification of 300 dairy cows into three phenotypic categories: high resistance, medium resistance, and susceptibility. Cows with an MSE value exceeding 1 × 10^−1^ or possessing one or no resistance genotypes were classified as susceptible. In contrast, cows with MSE values between 1 × 10^−2^ and 1 × 10^−1^ or having two resistance genotypes were deemed moderately resistant. Cows with MSE values below 1 × 10^−2^ or containing three or more resistance genotypes were categorized as highly resistant. Table A3 provides a summary of the resistance genotypes, which include the CA genotype of the NOD2 gene at the C>A locus, the CC genotype of the CXCR1 gene at the G>C locus, the AA genotype of the SPP1 gene at the G>A locus, and the GA genotype of the LF gene at the G>A locus. For instance, when the CA genotype at the NOD2 C>A locus is identified as resistant, the other loci are considered non-resistant genotypes. For example, as shown for NOD2 (CA) in the third row of Table 5), the MSE value would be 4.55 × 10^−1^, which exceeds 1 × 10^−1^, indicating that the dairy cow individual is susceptible.

#### 3.3.2. Genotyping Accuracy of KASP Kit for Recessive Mastitis in Dairy Cows

The results of testing 300 Holstein cows for resistance to occult mastitis using the kit revealed that 92 cows belonged to the high resistance category (Table 6). An analysis of the average somatic cell count (SCC) across the entire lactation period in the Dairy Herd Improvement (DHI) data for these 92 highly resistant individuals showed that three cows had an individual SCC exceeding 200,000/mL. Consequently, the accuracy rate for detecting high resistance with the kit was calculated as follows: [(92 − 3)/92] × 100% = 96.74%. Additionally, there were 148 cows classified as having moderate resistance. The average SCC over the entire lactation period for these moderately resistant cows indicated that six cows had an SCC either below 20 or above 500,000/mL, resulting in a resistance accuracy rate of [(148 − 6)/148] × 100% = 95.95%. In the DHI data for susceptible individuals, the average SCC over the entire lactation period revealed that three cows had an SCC below 500,000/mL. Therefore, the accuracy rate for detecting high resistance by the kit in this group was [(60 − 3)/60] × 100% = 95.00%. Overall, the average accuracy across the three groups was 95.90%, indicating that the KASP genotyping kit demonstrated high accuracy and can effectively be utilized to detect the resistance genome associated with recessive mastitis in dairy cows. The calculation formula is as follows:

(4) $$K=\frac{A-B}{A}\times 100\%$$

In the formula, K represents the resistance detection accuracy of the KASP kit, A represents the number of individual cows tested by genotype typing, and B represents the number of individual cows whose SCC and genotype typing results are inconsistent.

## 4. Discussion

Occult mastitis in dairy cows represents a significant constraint on the economic viability of dairy farms. This condition is characterized by the absence of overt abnormalities in the udder or milk, leading to an elevated somatic cell count (SCC) and reduced milk yield [62]. The incidence of occult mastitis is 15 to 40 times higher than that of clinical mastitis, and the absence of clear symptoms complicates its diagnosis [46]. Factors such as environmental conditions, the transmission of pathogenic microorganisms during milking, feeding management, genetics, and other variables can influence the susceptibility or resistance of dairy cows to mastitis [8,63,64]. Alterations in milk composition and the presence of drug residues associated with occult mastitis can adversely affect dairy product quality [65,66,67,68,69]. Furthermore, dairy consumption is often considered a key indicator of the living standards within a country or region [70]. The quality of raw milk is intrinsically linked to the flavor, sensory attributes, hygiene, and nutritional value of dairy products [71]. Consequently, enhancing the resistance of dairy cows to mastitis is crucial for ensuring the quality of dairy products. In recent years, there has been a growing body of research concerning genetic variations in dairy cows and their relationship to mastitis. The genetic enhancement of dairy cow breeds has significantly increased milk production [72]. However, as the genetic advancements in milk production accelerate, they are accompanied by health issues and a gradual rise in associated costs [42]. Research [73] has identified that the estimated genetic correlation between milk production and mastitis ranges from 0.07 to 0.33, with an average of approximately 0.20. This positive genetic correlation suggests that cows with higher genetic potential for milk production also possess a higher genetic predisposition for infection rates and mastitis. In the absence of selection against mastitis, the genetic increase in mastitis incidence is directly proportional to the genetic gains in milk production. Consequently, as the genetic enhancement of milk production accelerates at an unprecedented rate, the incidence of genetic predisposition to mastitis is also progressively increasing.

Occult mastitis in dairy cows is influenced by multiple genes, and focusing solely on the effect of a single gene or locus in relation to occult mastitis resistance makes it challenging to identify individuals with optimal resistance [42]. Sun et al. [61] proposed a method called multi-locus penetrance variance analysis of variance and systematically simulated its effectiveness. This method is highly suitable for analyzing potential mastitis resistance in dairy cows. Ma et al. [74] discovered that the aggregation of multiple resistance genes within the same breed not only broadens the spectrum of resistance but also enhances the level of resistance to certain strains. Typically, varieties possessing three or four resistance genes exhibit superior resistance compared to those containing no resistance genes or only one or two. The impact of genotype combinations is not merely additive; rather, it surpasses the effect of the most effective single genotype. When three genes are combined, the dominant genotype of a single gene remains the most effective combined genotype, and its effect exceeds that of the highest-performing single genotype [75]. In this study, genes associated with mastitis resistance in dairy cows were genotyped using KASP technology and correlated with somatic cell scores. Furthermore, multiple dominant genes were integrated through polygenic polymerization analysis, enabling the selection of individuals with optimal resistance to mastitis within dairy herds from the integrated dominant genomes.

### 4.1. The Influence of NOD2 Gene Polymorphism on Occult Mastitis in Dairy Cows

The NOD2 gene polymorphism has been previously identified as a susceptibility locus for human inflammatory bowel disease [76]. This gene is integral in initiating the inflammatory and subsequent immune responses. Chen et al. [6] conducted a transcriptome analysis to compare differentially expressed genes in the liver and spleen tissues of Holstein cattle and Yunnan Hump cattle. They discovered that genes associated with immune function, such as C1QB and NOD2, were up-regulated in Yunnan Hump cattle. The findings indicated that Yunnan Hump cattle exhibit high disease resistance, suggesting that the NOD2 gene may enhance the innate disease resistance of dairy cows. Wang et al. [77] identified two mutations in exon 12 of the NOD2 gene and investigated their association with production traits and genetic resistance to mastitis in Chinese Holstein and Simmental cattle. Their findings demonstrated a significant correlation between NOD2 polymorphism, mastitis incidence, and 305-day milk production. Specifically, allele B was associated with an increased somatic cell count, suggesting a potential link to mastitis susceptibility, whereas allele F was associated with a reduction in somatic cell count and an increase in milk production. Pant et al. [78] identified four mutations in the NOD2 gene, among which the SNP c.4500 polymorphism may influence the host response to mastitis, confirming the results of our study. This investigation identified a mutation in SNP c.4500C>A, revealing that individuals with the CA genotype had significantly lower cell scores compared to those with CC and AA genotypes.

### 4.2. The Influence of CXCR1 Gene Polymorphism on Occult Mastitis in Dairy Cows

The CXCR1 gene polymorphism has been predominantly linked to a range of human diseases, including ovarian cancer, hepatocellular carcinoma, and breast cancer, and is implicated in tumor proliferation, metastasis, and angiogenesis [79]. Gallegos-Arreola [80] demonstrated that the CXCR1 gene polymorphisms rs1008562, rs2234671, and rs3138060 are associated with an increased susceptibility to human breast cancer. Furthermore, Verbeke et al. [81] reported that CXCR1 polymorphism may influence the activity and concentration of polymorphonuclear neutrophils (PMN) in the mammary glands of dairy cows. Since the study by Youngerman et al. [82] in 2004, which identified the CXCR1 polymorphism GG variant at the +735 locus as being associated with a reduced incidence of occult mastitis, the CXCR1 gene has been regarded as a potential marker for mastitis in dairy cows. Additionally, Pushpa et al. [83] investigated the presence of CXCR1 gene polymorphism in Hardhenu (*Bos taurus* × *Bos indicus*) cattle and its correlation with clinical mastitis, reproductive disorders, and production performance traits, concluding that the CC genotype is more susceptible to clinical mastitis. Furthermore, individuals possessing the CC genotype exhibited superior milk production compared to those with CT and TT genotypes, suggesting a positive correlation between the C allele and milk yield. These findings hold significant implications for the genetic enhancement of Hardhenu (*Bos taurus* × *Bos indicus*) cattle. The integration of identified CXCR1 gene polymorphisms into current molecular markers may improve both disease resistance and milk production performance in cows. Galvao et al. [84] reported that cows with the CC genotype at the CXCR1+777 locus demonstrated a lower somatic cell count (SCC) in comparison to those with GG and GC genotypes, which aligns with the results of the present study [85].

### 4.3. The Influence of SPP1 Gene Polymorphism on Occult Mastitis in Dairy Cows

Secreted pyrophosphoprotein-1 (SPP1) is an acidic glycoprotein that is secreted and possesses multiple biological functions. It is currently recognized as a significant cytokine and an extracellular integrin-binding protein, playing a crucial role in both inflammation and homeostasis. Maria et al. [86] conducted an association analysis on the polymorphism of the SPP1 gene in Salda sheep, revealing that the rs161844011 locus in exon 7 is correlated with somatic cell score (SCS). Mello et al. [87] investigated the relationship between SPP1 gene polymorphism and milk yield in Girorando dairy cows, finding that cows at 305 days of lactation carrying the T allele at the g.8514 locus exhibited higher milk production, although this increase did not achieve statistical significance. Furthermore, the polymorphism of the SPP1 gene has been found to be significantly correlated with lactation persistence in dairy cows, with the G allele identified as the dominant variant. Liang [88] reported that individuals with the CC genotype and the C allele of the SPP1 gene at rs11730582 demonstrated a reduced risk of developing breast cancer. Additionally, SUN et al. [89] identified two single nucleotide polymorphisms (SNPs) within the intron region of the SPP1 gene. In comparison to the AA genotype, the SPP1-g.50265 G>A loci were AG and GG genotypes exhibited significantly higher milk yield and milk urea nitrogen content. Conversely, the SPP1-g.50315 C>T loci CT and TT genotypes demonstrated significantly elevated milk production and milk urea nitrogen content, with a higher fat percentage compared to the CC genotype. De et al. [87] reported that genetic variations in the SPP1 gene could influence milk yield and local mammary gland immunity in dairy cows, with different genotypes significantly affecting somatic cell scores. Huboby, H.A. et al. [90] identified a single nucleotide polymorphism(SNP) at the G>A position in the exon 5 promoter region of the SPP1 gene, specifically at the P/4187 location, and noted significant differences in the daily milk yield of ewes (*p* ≤ 0.05), this study indicates a correlation between SNPs and certain economic traits in local Awassi sheep, which can be leveraged in breeding and improvement programs. Bissonnette et al. [91] investigated the association between SNPs in the promoter region of the SPP1 gene and somatic cell scores in Holstein dairy cows. Their findings indicated that individuals possessing the AA genotype at the SPP1-1303 locus exhibited significantly lower somatic cell scores compared to those with GG and GA genotypes. This suggests a significant association between SPP1 gene polymorphisms and recessive mastitis in dairy cows, aligning with the findings of the present study.

### 4.4. The Influence of LF Gene Polymorphism on Occult Mastitis in Dairy Cows

Lactoferrin(LF) genes exhibit significant polymorphic variation, particularly within and adjacent to transcription factor binding sites [92]. These polymorphisms are crucial as they may impact gene expression levels during immune system activation in response to infection. Zheng et al. [93] demonstrated through promoter deletion analysis that the sequence at the SNP-543 site is sufficient to activate the core promoter, thereby modulating the transcription of bovine lactoferrin. Nowier et al. [94] identified a mutation (G/C) at the SNP+32 site within the LF gene promoter fragment in Holstein cows, revealing that individuals with the CC genotype exhibited significantly higher milk protein content compared to those with the GG genotype. Carvajal et al. [95] established an association between SNPc.-28 A>C mutations in the LF gene promoter region and mastitis resistance in dairy cows. Furthermore, Halloran et al. [96] identified three single nucleotide polymorphisms (SNPs) within the bovine lactoferrin promoter region, which were previously linked to reproductive performance and somatic cell count (SCC). Lionel et al. [97] demonstrated that the LF genotype with a low somatic cell count (SCC) positively influences milk protein, lactose, and overall milk yield in dairy cows. Pawlik et al. [98] identified three single nucleotide polymorphisms (SNPs) in the LF LF-926 GG, LF+32 GG and GC, LFex4 AA, LFex9 TT as excellent markers for dairy performance in Polish Holstein cows. Li et al. [99] conducted variance analysis, and a number of comparative studies revealed that the LF gene g.4388 G>C mutation site in Holstein cattle significantly affects somatic cell score (SCS) (*p* < 0.01). Specifically, individuals with the GC genotype exhibited significantly lower SCS values compared to those with GG and CC genotypes (*p* < 0.01), and this mutation also had significant effects on daily milk yield and measured daily milk fat percentage (*p* < 0.05). These findings align with the results of the present study, which identified a significant association between the LF-190 G>A polymorphism and SCS (*p* < 0.05), with the GA genotype displaying lower SCC than both the GG and AA genotypes. These results suggest that LF gene polymorphism may be linked to occult mastitis resistance in dairy cows.

In the post-genomic era, the competitive allele-specific PCR genotyping technology developed by LGC enables accurate biallelic judgments on SNPs and insertions/deletions (InDels) at specific sites. Tao et al. [100] utilized KASP technology to conduct typing and correlation analysis on 26 mutation sites within the WWC2, ARHGEF9, SLK, GAB3, and FSHR genes. Their findings indicated that the loci WWC2 (g.14962207 C>T), SLK (g.27108855 G>A), ARHGEF9 (g.48271079 C>A), and FSHR (g.80789180 T>G) have potential as molecular markers for assessing variations in sheep reproductive performance. Furthermore, Chang et al. [40] compared KASP assay results with those obtained from PCR-RFLP assays on 107 racing pigeon samples, demonstrating complete (100%) concordance between the two methodologies. Given the rapid, reliable, efficient, and cost-effective nature of KASP analysis, it presents substantial advantages over PCR-RFLP. Ilie et al. [41] employed competitive allele-specific PCR (KASP) to genotype 298 cattle, including 250 Romanian spotted cattle and 48 Romanian brown cattle and utilized the chi-square test to examine correlations with clinical mastitis. Genetic correlation studies confirmed that the rs110124025 SNP in BOLA-DRB3 is associated with mastitis resistance, indicating that this SNP could serve as a potential candidate marker for selecting mammary gland health in Romanian dairy cows. Yang et al. [101] were the first to apply KASP technology to establish and optimize a detection method for ABO blood groups in rhesus and cynomolgus monkeys. This method is distinguished by its simplicity, speed, cost-effectiveness, and reliability compared to traditional serological or sequencing techniques. This experiment extends the findings of multi-gene aggregation studies by utilizing Kompetitive Allele-Specific PCR (KASP) technology to effectively classify and identify recessive mastitis resistance genes in dairy cows, achieving an individual cow genotyping accuracy of 100%. Through the analysis of diverse genotype combinations, the study enables the assessment of individual dairy cows’ resistance to occult mastitis.

## 5. Conclusions

In this study, multi-locus penetrance variance analysis (MPVA) was employed to assess the resistance and susceptibility of various genotype combinations to occult mastitis, the genotype combination SPP1(AA)-CXCR1(CC)-NOD2(CA)-LF(GA) was identified as the dominant genotype conferring resistance to occult mastitis. These genotype combinations can serve as molecular markers for the identification of resistance genes associated with occult mastitis in dairy cows; for using KASP technology, a genomic detection kit was successfully developed for identifying resistance to occult mastitis in dairy cows. This kit demonstrated a 100% accuracy rate for individual genotyping and a 95.90% accuracy rate for population resistance detection. It facilitates the detection of occult mastitis resistance in individual dairy cows at any stage of feeding, thereby enabling the removal of susceptible individuals and contributing to the overall improvement of the cattle herd.

## Figures and Tables

**Table 1:** Primer sequences for KASP assays.

Mutation Site	Fluorescent Signal	Primer	Sequence (5′-3′)
NOD2 C>A site	FAM	F1	GAGACACTTGGAGAGAATGGAGC
	HEX	F2	GGAGACACTTGGAGAGAATGGAGA
	/	R	CAGCGTGGAGTTGTAAGTTATAGAGA
CXCR1 G>C site	FAM	F1	CCAAATGGGGCACAAGCAC
	HEX	F2	CCAAATGGGGCACAAGCAG
	/	R	GCCCTCATGAGGGTGTCCG
SPP1 G>A site	FAM	F1	CAGATGCTCTCCACCTACACAGG
	HEX	F2	CCAGATGCTCTCCACCTACACAGA
	/	R	TTCTGTGACCACAAAACCAGAGG
LF G>A site	FAM	F1	GGACAACTACAAGGTCTACAACACA
	HEX	F2	GGACAACTACAAGGTCTACAACACG
	/	R	CTTCTTGGTCCTAATGCCCTCAGA

**Table 2:** The composition of the KASP genotyping kit.

Composition	Quantity and Specifications
SNP Primer Mix: Upstream primer F1, upstream primer F2, universal downstream primer	2.2 mL each
2× KASP Master mix	2.5 mL each
ddH_2_O	1.5 mL each

**Table 3:** PCR reaction system.

Composition	96-Well Plates (μL)	384-Well Plates (μL)
DNA	1	1
SNP Primer Mix	0.14	0.07
2× KASP Master mix	4	2
ddH_2_O	2.86	0.93
total volume	8	4

**Table 4:** PCR amplification system.

Steps	Process	Temperature/°C	Time	Number of Cycles
1	Pre-degeneration	95	10 min	1 cycle
2	Denaturation	95	20 s	10 cycles
	Annealing/Extension	61–55 °C (drop 0.6 °C per cycle)	45 s	
3	Denaturation	95	20 s	38 cycles
	Annealing/Extension	55	45 s	
4	End	25	Forever	1

**Table 5:** Classification of resistance to occult mastitis in 300 dairy cows.

Genotype	MSE Value	Somatic Cell Count (Ten Thousand/mL)
SPP1(AA)	5.45 × 10^−1^	Susceptible type
CXCR1(CC)	3.89 × 10^−1^	Susceptible type
NOD2(CA)	4.55 × 10^−1^	Susceptible type
LF(GA)	3.25 × 10^−1^	Susceptible type
SPP1(AA)- CXCR1(CC)	7.78 × 10^−2^	Intermediate-resistant type
SPP1(AA)- NOD2(CA)	7.56 × 10^−2^	Intermediate-resistant type
CXCR1(CC)- NOD2(CA)	6.56 × 10^−2^	Intermediate-resistant type
LF(GA)-SPP1(AA)	4.56 × 10^−2^	Intermediate-resistant type
LF(GA)-CXCR1(CC)	4.12 × 10^−2^	Intermediate-resistant type
LF(GA)-NOD2(CA)	5.26 × 10^−2^	Intermediate-resistant type
CXCR1(CC)-SPP1(AA)-NOD2(CA)	5.52 × 10^−3^	Highly-resistant type
CXCR1(CC)-SPP1(AA)-LF(GA)	4.52 × 10^−3^	Highly-resistant type
SPP1(AA)-NOD2(CA)-LF(GA)	6.25 × 10^−3^	Highly-resistant type
CXCR1(CC)-NOD2(CA)-LF(GA)	7.15 × 10^−3^	Highly-resistant type
CXCR1(CC)-SPP1(AA)-NOD2(CA)-LF(GA)	3.23 × 10^−4^	Highly-resistant type

**Table 6:** Based on the KASP kit and the average somatic cell count throughout the entire lactation period as recorded in DHI data, the study identified individuals with susceptibility, high antibody levels, and medium antibody levels. Subsequently, the accuracy of the kit was evaluated.

Grouping	Kit Results	Somatic Cell Count (Million per Milliliter)			Accuracy
		<20	20–50	>50	
High resistance/each	92	89	3	0	(89/92) × 100% = 96.74%
Medium resistance/each	148	4	142	2	(142/148) × 100% = 95.95%
Susceptible/each	60	1	2	57	(57/60) × 100% = 95.00%

## Data Availability

The original contributions presented in this study are included in the article. Further inquiries can be directed to the corresponding author.

## Abbreviations

SCC	Somatic Cell Count
KASP	Kompetitive Allele-Specific
MPVA	Multi-Locus Penetrance Variance Analysis
DHI	Dairy Herd Improvement
SNP	Single Nucleotide Polymorphisms
NOD2	Nucleotide-binding Oligomerization Domain Protein 2
CXCR1	CXC-chemokine receptor 1
SPP1	Secreted Pyrophosphoprotein-1
LF	Lactoferrin
InDels	Insertions/Deletions
PIC	Polymorphism Information Content
MSE	Minimum Mean Squared Error
SCS	Somatic Cell Score
KBP	Kilobase Pairs
LPS	Lipopolysaccharides

## Appendix A

**Table A1:** Genotype frequencies and allele frequencies and main diversity indices [expected (He) and observed (Ho) heterozygosity, the effective number of alleles (Ne), polymorphism information content (PIC)] for the polymorphic SNPs in Holstein cows.

SNPs	Genotype Frequency			Allele Frequency		*Ho*	*He*	*Ne*	*PIC*	*p*-Value
NOD2c.4500 C>A	CC 22.22% (n = 86)	CA 52.59% (n = 205)	AA 25.16% (n = 98)	C 48.52%	A 51.48%	0.50	0.50	1.86	0.38	0.65
CXCR1+777 G>C	GG 27.41% (n = 107)	GC 24.44% (n = 95)	CC 48.15% (n = 187)	G 60.37%	C 39.63%	0.52	0.48	1.72	0.36	0.53
SPP1-1301 G>A	GG 22.22% (n = 86)	GA 33.33% (n = 130)	AA 44.44% (n = 173)	G 21.11%	A 78.89%	0.67	0.33	1.54	0.25	0.84
LF-190 G>A	GG 40.74% (n = 158)	GA 41.48% (n = 161)	AA 17.78% (n = 70)	G 61.21%	A 38.79%	0.62	0.38	1.74	0.37	0.51

*PIC* < 0.25, low polymorphism; 0.25 < *PIC* < 0.5, moderate polymorphism; *PIC* > 0.5, highly polymorphic.

**Table A2:** Correlation analysis of NOD2C4500, CXCR1+777, SPP1-1301, and LF-190 genotypes with somatic cell score in Holstein cows. ^a,b^: Column means with different letter superscripts differ significantly at *p* < 0.05 within the same SNP, while no significant differences were observed among those with the same letters (*p* > 0.05).

Locus	Genotype	Number of Records	*p*-Value	Somatic Cell Score
NOD2c.4500 C>A	CC	86	0.038	5.58 ± 2.17 ^a^
	AA	205		5.26 ± 2.24 ^a^
	CA	98		3.68 ± 2.38 ^b^
CXCR1+777 G>C	GG	107	0.045	4.7 ± 2.26 ^a^
	CG	95		4.71 ± 2.55 ^a^
	CC	187		3.25 ± 1.35 ^b^
SPP1-1301 G>A	GA	86	0.013	5.83 ± 1.79 ^a^
	GG	130		6.76 ± 1.13 ^a^
	AA	173		2.71 ± 2.44 ^b^
LF-190G>A	GG	158	0.025	6.54 ± 2.45 ^a^
	GA	161		5.15 ± 1.85 ^a^
	AA	70		4.56 ± 2.45 ^b^

**Table A3:** The result of combinations of genotypes in the condition of multi-locus.

Gene				MSE Value	Somatic Cell Count (Ten Thousand/mL)
SPP1 (GG)	CXCR1 (CG)	NOD2 (CC)	LF (GG)	1.00 × 10^−4^	58.87 ± 5.92
SPP1 (GG)	CXCR1 (CC)	NOD2 (CC)	LF (GG)	9.98 × 10^−3^	52.10 ± 4.72
SPP1 (GG)	CXCR1 (GG)	NOD2 (CC)	LF (GG)	1.00 × 10^−4^	56.86 ± 5.63
SPP1 (AG)	CXCR1 (CG)	NOD2 (CC)	LF (GG)	1.02 × 10^−2^	55.94 ± 6.58
SPP1 (AG)	CXCR1 (CC)	NOD2 (CC)	LF (GG)	1.05 × 10^−−3^	48.17 ± 5.38
SPP1 (AG)	CXCR1 (GG)	NOD2 (CC)	LF (GG)	1.02 × 10^−4^	55.93 ± 6.29
SPP1 (AA)	CXCR1 (CG)	NOD2 (CC)	LF (GG)	1.01 × 10^−3^	52.82 ± 7.23
SPP1 (AA)	CXCR1 (CC)	NOD2 (CC)	LF (GG)	1.03 × 10^−2^	25.05 ± 6.03
SPP1 (AA)	CXCR1 (GG)	NOD2 (CC)	LF (GG)	1.00 × 10^−3^	52.81 ± 6.94
SPP1 (GG)	CXCR1 (CG)	NOD2 (CA)	LF (GA)	1.02 × 10^−2^	35.56 ± 4.91
SPP1 (GG)	CXCR1 (CC)	NOD2 (CA)	LF (GA)	1.58 × 10^−1^	19.70 ± 3.71
SPP1 (GG)	CXCR1 (GG)	NOD2 (CA)	LF (GA)	1.61 × 10^−2^	29.46 ± 4.62
SPP1 (AG)	CXCR1 (CG)	NOD2 (CA)	LF (GA)	6.21 × 10^−2^	28.54 ± 5.57
SPP1 (AG)	CXCR1 (CC)	NOD2 (CA)	LF (GA)	5.78 × 10^−2^	19.77 ± 4.37
SPP1 (AG)	CXCR1 (GG)	NOD2 (CA)	LF (GA)	6.37 × 10^−2^	23.53 ± 5.28
SPP1 (AA)	CXCR1 (CG)	NOD2 (CA)	LF (GA)	6.45 × 10^−2^	29.42 ± 6.22
SPP1 (AA)	CXCR1 (CC)	NOD2 (CA)	LF (GA)	1.01 × 10^−4^	17.45 ± 5.02
SPP1 (AA)	CXCR1 (GG)	NOD2 (CA)	LF (GA)	2.49 × 10^−1^	25.41 ± 5.93
SPP1 (GG)	CXCR1 (CG)	NOD2 (AA)	LF (AA)	1.32 × 10^−4^	48.57 ± 4.35
SPP1 (GG)	CXCR1 (CC)	NOD2 (AA)	LF (AA)	1.69 × 10^−3^	50.26 ± 5.26
SPP1 (GG)	CXCR1 (GG)	NOD2 (AA)	LF (AA)	1.02 × 10^−4^	45.31 ± 5.14
SPP1 (AG)	CXCR1 (CG)	NOD2 (AA)	LF (AA)	1.56 × 10^−4^	46.94 ± 6.52
SPP1 (AG)	CXCR1 (CC)	NOD2 (AA)	LF (AA)	4.25 × 10^−3^	50.45 ± 2.56
SPP1 (AG)	CXCR1 (GG)	NOD2 (AA)	LF (AA)	8.56 × 10^−4^	49.25 ± 6.23
SPP1 (AA)	CXCR1 (CG)	NOD2 (AA)	LF (AA)	3.25 × 10^−4^	50.05 ± 6.22
SPP1 (AA)	CXCR1 (CC)	NOD2 (AA)	LF (AA)	1.95 × 10^−2^	36.29 ± 5.13
SPP1 (AA)	CXCR1 (GG)	NOD2 (AA)	LF (AA)	5.26 × 10^−3^	54.58 ± 3.56
